# Impact of Isolated Tricuspid Valve Repair on Right Ventricular Remodelling in an Adult Congenital Heart Disease Population

**DOI:** 10.3389/fcvm.2017.00021

**Published:** 2017-04-28

**Authors:** Roberto Marsico, Vito Domenico Bruno, Pierpaolo Chivasso, Anna Baritussio, Filippo Rapetto, Gustavo A. Guida, Umberto Benedetto, Massimo Caputo

**Affiliations:** ^1^School of Clinical Sciences, Bristol Heart Institute, University of Bristol, Bristol, UK; ^2^Cardiovascular Magnetic Resonance Unit, NIHR Bristol Cardiovascular Biomedical Research Unit, Bristol Heart Institute, University of Bristol, Bristol, UK

**Keywords:** tricuspid valve repair, adult congenital heart disease, echocardiography, right ventricular dysfunction, ventricular reverse remodelling

## Abstract

**Background:**

Surgical repair of isolated congenital tricuspid valve (TV) disease is rare with no well-defined indication and outcomes. Moreover, the role of right ventricle (RV) in this context has not yet been investigated.

**Objectives:**

We sought to assess the impact of congenital TV repair on cardiac remodelling and clinical–functional status and the importance of the RV function in an adult congenital heart disease (ACHD) population.

**Methods and results:**

From January 2005 to December 2015, 304 patients underwent TV surgery in our centre. Of these, 27 (ACHD) patients had isolated TV repair. Patients were evaluated with preoperative and postoperative transthoracic echocardiogram. Survival rate has been investigated with a mean clinical follow-up (FU) of 3.7 ± 2.3 years, whereas the mean echocardiographic FU was 2.9 ± 1.8 years. The clinical and functional status of patients showed a statistically significant improvement after the surgical repair in terms of New York Heart Association class (66.7 vs 7.4%; *p* < 0.01), clinical signs of heart failure (29.6 vs 7.4%; *p* < 0.01), and left ventricular function (14.8 vs 7.4%; *p* < 0.01). The RV and right atrium diameter were significantly reduced after surgery (5.15 ± 1.21 vs 4.32 ± 1.16; *p* < 0.01) and (44.7 ± 16.7 vs 26.7 ± 9.2; *p* < 0.01), respectively. The degree of postoperative pulmonary hypertension was also significantly reduced (40.7 vs 7.4%; *p* < 0.01). The survival rate was 96.3% at 1 year and 93.7% at 5 years. One patient (3.7%) had early failure of the tricuspid repair requiring a reoperation.

**Conclusion:**

Isolated TV repair for adult congenital disease significantly improved patients’ clinical and functional status and allowed right ventricular remodelling and functional improvement.

## Introduction

Isolated severe congenital tricuspid regurgitation (TR) is an uncommon condition, most frequently associated with a variety of concomitant heart diseases. Regardless of the presentation, the surgical management is similar (1, 2). To date, a limited body of evidence is available on the outcomes of this subset of patients, and the published surgical guidelines are not clear in terms of surgical procedure and timing for surgery (3, 4). Furthermore, the negative impact of TR on long-term prognosis has been largely demonstrated (5), and it has been advocated that surgery should be considered before the development of right ventricle (RV) systolic dysfunction (6); in fact, these patients frequently have evidence of right heart failure and its concomitant complications (7). Patients are rarely referred for isolated surgical tricuspid valve (TV) repair, and most repairs are done in the context of other planned cardiac operations (4). In this study, we sought to assess the impact of isolated TV repair on cardiac remodelling and clinical–functional status, and specifically its effect on the RV function in an adult congenital heart disease (ACHD) population.

## Materials and Methods

The study was conducted in accordance with the principles of the Declaration of Helsinki. The local audit committee approved the study, and the requirement for individual patient consent was waived.

**Figure 1:**
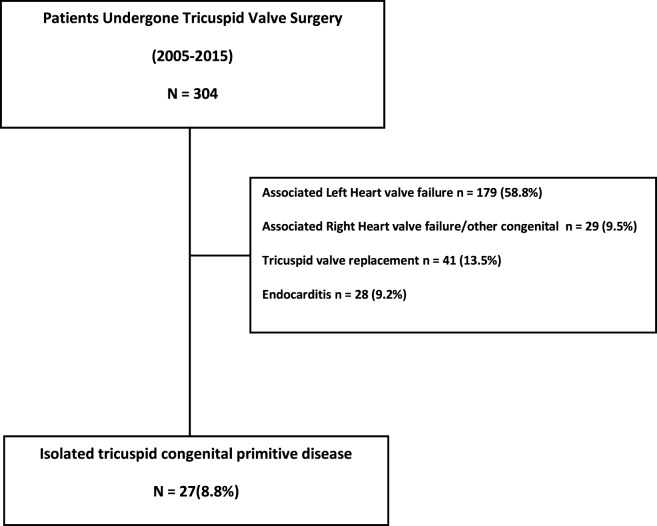
**Patient selection and exclusion criteria**.

From January 2005 to December 2015, a total of 304 adult patients underwent TV repair or replacement at Bristol Heart Institute. Patients with concomitant left heart valve diseases and/or other cardiac congenital diseases, those who underwent TV replacement and those with endocarditis were excluded (Figure 1) from our analysis. Of these, 179 (58.8%) had a functional TR secondary to left heart valve failure (mitral regurgitation and/or aortic valve disease); 29 (9.5%) patients had TR associated with pulmonary valve dysfunction; 28 patients (9.2%) had isolated TV regurgitation secondary to endocarditis. Forty-one patients (13.5%) had TV replacement with prosthesis.

The remaining 27 (8.8%) were defined as ACHD patients undergoing surgical TV repair for isolated TR.

### Clinical and Functional Assessment

The clinical conditions were assessed following the classification proposed by the New York Heart Association (NYHA) and Canadian Cardiovascular Society (CCS). Both preoperatively and at the end of follow-up (FU), patients were assigned to a correlated class. Clinical signs and symptoms of RV failure and onset of newly discovered cardiac arrhythmias were regularly assessed during FU outpatient clinics. Short-term outcomes were derived from clinical notes: acute kidney injury was defined as an increase, during admission, of over 50% in serum creatinine compared to preoperative values, as previously recommended (8); acute renal failure was defined as the need for perioperative kidney replacement therapy. Cerebrovascular accident (CVA) was defined on the basis of a focal or global neurological impairment at physical examination or at CT scan/magnetic resonance imaging. Deep wound infection was defined as a surgical site-related infection affecting the median sternotomy wound and requiring antibiotics and/or surgical re-exploration. Short- and long-term survival data have been collected from the National Institute for Cardiovascular Outcome Research. The 30-day mortality was defined as death from any cause during the first 30 days following surgery.

### Echocardiographic Evaluation

All patients underwent transthoracic echocardiography preoperatively, within 2 weeks after the operation and at FU. The following parameters were assessed: left ventricular ejection fraction (LVEF), left ventricular internal diastolic diameter, left ventricular internal systolic diameter, thickness of the interventricular septum in diastole, diameter of the posterior wall, RV functional assessment (RV failure), right ventricular internal diastolic diameter, right ventricle dilatation (RV dilatation), area of left atrium (LA area), area of right atrium (RA area), pulmonary artery pressure (PAP), grade of pulmonary hypertension, grade of TV regurgitation, tricuspid annular plane systolic excursion (tapse), and presence of haepatic veins backflow.

### Statistical Analysis

Data are presented as mean ± 1 SD for continuous variables or as number and percentages for dichotomous variables. Continuous variables were tested for normality using the Kolmogorov–Smirnov test and then compared between groups with unpaired Student’s *t*-test if normally distributed or Mann–Whitney *U* test if not normally distributed. In the case of dichotomous or categorical variables, Pearson chi-square or Fisher exact tests were used as appropriate. Overall long-term survival was estimated by Kaplan–Meier curve. All tests were two-sided with the alpha level set at 0.05 for statistical significance. The statistical analysis was computed using R version 3.0.2 for Windows (R Foundation for Statistical Computing, Vienna, Austria).

## Results

Patient characteristics and preoperative variables are summarised in Table 1. Mean age was 51.62 ± 14.4 years (range 17–73 years) and 18 patients (66.7%) were female. The mean clinical FU was 3.7 ± 2.3 years, while the mean echocardiographic FU time was 2.9 ± 1.8 years. Table 2 shows the AHDC population including the TV aetiopathology for each patient. Ebstein anomaly was diagnosed in 14 (51.8%) patients, whereas the remaining 13 patients (48.1%) were affected by non-Ebstein tricuspid dysplasia. Thirteen patients (48.1%) had right ventricular failure at the time of surgery and 22 (81.4%) had dilated RV. Four patients were found to have reduced left ventricular function (14.7%). Of our cohort of patients, 33.3% were diagnosed with preoperative atrial fibrillation, and one patient (3.7%) had a permanent pace maker for atrioventricular block. Ascites was present in almost one-third of the patients, while two-thirds of the patients were in NYHA class III or IV (Table 1). Mean logistic Euroscore was 5.39 ± 8.3 and three (11.1%) patients had a previous cardiac surgery operation.

**Table 1:** **Preoperative characteristics of patients with isolated tricuspid repair (*n* = 27)**.

Characteristic	Patients
Age: years	51.62 ± 14.39
Female gender	18 (66.7%)
Reduced LVEF (moderate to severe)	4 (14.7%)
RV failure (moderate to severe)	13 (48.1%)
RV dilatation	22 (81.4%)
Renal impairment	0
COPD	5 (18.5%)
Diabetes	2 (7.4%)
Hypertension	8 (29.6%)
Previous CVA	3 (11.1%)
TIA	1 (3.7%)
Smoking history	
Current	0
Ex-smoker	8 (29.6%)
PVD	0
Reoperation	3 (11.1%)
Number of previous heart operations	
2	1 (3.7%)
1	2 (7.4%)
Ebstein disease	14 (51.8%)
Tricuspid dysplasia	13 (48.1%)
Euro score: %	5.39 ± 8.30
Min: %	1.52
Max: %	44.82
Heart rhythm	
Sinus rhythm	17 (62.9%)
AF	9 (33.3%)
AV block (paced)	1 (3.7%)
Ascites	8 (29.6%)
NYHA class 3 or 4	18 (66.7%)
CCS class 3 or 4	6 (22.2%)

*Data are presented as mean ± SD, median (range), or *n* (%)*.

*LVEF, left ventricular ejection fraction; RV, right ventricle; COPD, chronic obstructive pulmonary disease; CVA, cardiovascular accidents; TIA, transient ischaemic attack; PVD, peripheral vascular disease; NYHA, New York Heart Association; CCS, Canadian Cardiovascular Society*.

*Data are expressed as number of events and percentages or otherwise expressed*.

**Table 2:** **Preoperative classification of congenital heart disease in patients with isolated tricuspid repair (*n* = 27)**.

Characteristic	Patients
Ebstein disease	14 (51.8%)
Non-Ebstein TR	13 (48.1%)
– Previous congenital heart surgery in childhood	3 (11.1%)
ASD	1 (3.7%)
TOF	1 (3.7%)
VSD	1 (3.7%)
– Tricuspid dysplasia associated PFO-small ASD	4 (14.8%)
– TV delamination defect/Ebsteinoid anomaly	6 (22.2%)

*Data are presented as mean ± SD, median (range), or *n* (%)*.

*ASD, atrial septal defect; TOF, tetralogy of fallot; VSD, ventricular septal defect; TR, tricuspid regurgitation; PFO, patent foramen ovale; TV, tricuspid valve*.

*Ebsteinoid anomaly (9): variable field of disease associated with TV leaflets’ failure of delamination*.

*Data are expressed as number of events and percentages or otherwise expressed*.

Intraoperative and postoperative findings are shown in Table 3. Nine patients (33.3%) underwent Cone procedure (http://www.ctsnet.org/article/cone-reconstruction-tricuspid-valve-repair-ebstein-anomaly), seven patients (25.9%) had annuloplasty ring and the remaining patients (40.7%) had other types of repair (De Vega procedure, Alfieri Stitch procedure and/or commissuroplasty/plication). Most patients had isolated TV surgery; concomitant ASD closure was performed in four (14.8%) patients; antiarrhythmic surgery such as MAZE procedure was also performed in four (14.8%) patients. Mean cardiopulmonary bypass time was 104.9 ± 44.7 min, and mean cross-clamp time was 66.3 ± 32.4 min. In one case (3.7%), with Ebstein disorder, it was necessary to re-operate during the postoperative course for failure of TV repair; this patient eventually underwent TV replacement. One patient (3.7%) suffered a postoperative deep wound infection (mediastinitis), which required surgical re-exploration: this condition deteriorated to postoperative sepsis and subsequent death. No patient experienced episodes of cerebral ischaemia (CVA) or renal impairment in the perioperative period.

**Table 3:** **Operative and postoperative characteristics of patients with isolated tricuspid repair (*n* = 27)**.

Characteristic	Patients
Tricuspid valve procedure	
Cone	9 (33.3%)
Annuloplasty ring	7 (25.9%)
Alfieri–plication–commissuroplasty	3 (11.1%)
Others	8 (29.6%)
PFO-small ASD closure	4 (14.8%)
MAZE	4 (14.8%)
CPB time (min)	104.96 ± 44.72
Cross-clamp time (min)	66.29 ± 32.41
Return to theatre	1 (3.7%)
Repair failure	1 (3.7%)
Deep wound infection	1 (3.7%)
New CVA	0
Post op dialysis	0
Length of hospital stay (days)	14.59 ± 11.30
Min (days)	5
Max (days)	53

*Data are presented as mean ± SD, median (range), or *n* (%)*.

*PFO, patent foramen ovale; ASD, atrial septal defect; CPB, cardio pulmonary bypass; CVA, cardiovascular accidents*.

*Data are expressed as number of events and percentages or otherwise expressed*.

### Follow-up

#### Clinical and Functional Results

The clinical and functional status of patients assessed according to the NYHA and CCS classification shows a statistically significant improvement: 66.7% patients in NYHA class III-IV preoperatively vs 7.4% at FU (*p* < 0.01) and 22.2% in CCS class III–IV preoperatively vs 3.7% at FU (*p* < 0.01). Also, our study demonstrated a significant reduction of clinical signs of heart failure such as ascites and peripheral oedema (29.6 vs 7.4%; *p* < 0.01). These data are shown in Table 4.

**Table 4:** **Clinical and functional assessment of patients with isolated tricuspid repair (*n* = 27)**.

Characteristic	Preoperative	Follow-up	*p*-Value
NYHA 3–4	18 (66.7%)	2 (7.4%)	<0.01
CCS high class	6 (22.2%)	1 (3.7%)	<0.01
Heart rhythm			
NSR	17 (62.9%)	18 (74%)	0.1
AF	9 (33.3%)	8 (29.6%)	0.1
Paced	1 (3.7%)	1 (3.7%)	0.1
Ascites p. oedema	8 (29.6%)	2 (7.4%)	<0.01

*Data are presented as mean ± SD, median (range), or *n* (%)*.

*NYHA, New York Heart Association; CCS, Canadian Cardiovascular Society; NSR, normal sinus rhythm; AF, atrial fibrillation*.

*Data are expressed as number of events and percentages or otherwise expressed*.

#### Echocardiographic Evaluations

Table 5 shows the preoperative and postoperative echocardiographic evaluation, with a mean FU echocardiographic time of 2.9 ± 1.8 years. There was a marked improvement in left ventricular function (14.8 vs 7.4%; *p* < 0.01), while no significant differences were found regard to the left ventricular diameters and wall thickness. Similarly, no significant differences were found concerning the left atrial volumes. At FU, all patients showed a significant reduction in right ventricular diameters (5.15 ± 1.21 vs 4.32 ± 1.16 cm; *p* < 0.01). We also observed a different remodelling in right atrial geometry (44.7 ± 16.7 vs 26.7 ± 9.2 cm^2^; *p* < 0.01). Regarding the functionality of TV, a clear reduction of valvular regurgitation has been observed at FU. Furthermore, TR significantly improved both in patients with moderate (22.2 vs 7.4%; *p* = 0.04) and severe baseline TR (77.7 vs 3.7%; *p* < 0.01). In addition, the pulmonary pressure (38.9 ± 7.8 vs 33.3 ± 5.5 mmHg; *p* = 0.03) and the degree of pulmonary hypertension (40.7 vs 7.4%; *p* < 0.01) were significantly reduced. A net reduction was also noticed in the number of patients with haepatic veins backflow (40.7 vs 7.4%; *p* < 0.01). On the other hand, TAPSE values dropped significantly in this period of evaluation (1.9 ± 0.85 vs 1.2 ± 0.36 mm; *p* < 0.01).

**Table 5:** **Echocardiographic evaluations of patients with isolated tricuspid repair (*n* = 27)**.

Characteristic	Preoperative	Follow-up	*p*-Value
Reduced left ventricular ejection fraction (mod-severe)	4 (14.8%)	2 (7.4%)	<0.01
LVIDD (cm)	4.18 ± 0.79	4.34 ± 0.70	0.1
LVISD (cm)	2.76 ± 0.71	2.87 ± 0.58	0.22
IVSD (cm)	0.93 ± 0.17	0.98 ± 0.16	0.1
PWD (cm)	0.89 ± 0.13	0.94 ± 0.14	0.1
RV failure (mod-severe)	13 (48.1%)	8 (29.6%)	0.1
RV dilatation	22 (81.4%)	11 (40.7%)	<0.01
Right ventricular internal diastolic diameter (cm)	5.15 ± 1.21	4.32 ± 1.16	<0.01
LA area (cm^2^)	21.3 ± 7.6	22.4 ± 8.39	0.57
RA area (cm^2^)	44.7 ± 16.7	26.7 ± 9.2	<0.01
Tricuspid regurgitation			
Moderate	6 (22.2%)	2 (7.4%)	0.04
Severe	21(77.7%)	1 (3.7%)	<0.01
Haepatic veins backflow	11(40.7%)	2 (7.4%)	<0.01
PAP (mmHg)	38.91 ± 7.88	33.37 ± 5.5	0.03
TAPSE (mm)	1.91 ± 0.85	1.26 ± 0.36	<0.01
P.Hypertension (mod-severe)	11(40.7%)	2 (7.4%)	<0.01

*Data are presented as mean ± SD, median (range), or *n* (%)*.

*LV, left ventricle; RV, right ventricle; LVIDD, left ventricular internal dimension in diastole; LVISD, left ventricular internal end-systolic dimension; IVSD, interventricular septum in diastole; PWD, posterior wall dimensions; RV, right ventricle; LA, left atrium; RA, right atrium; PAP, pulmonary arterial pressure; TAPSE, tricuspid annular plane systolic excursion; P.Hypertension, pulmonary hypertension*.

*Data are expressed as number of events and percentages or otherwise expressed*.

In Table 6, we reported the results of a sub-analysis that was conducted on the preoperative and postoperative echocardiographic evaluation in the Ebstein population. A significant improvement was found in left ventricular function and on the left ventricular diameters (3.64 ± 0.4 vs 4.0 ± 0.5 cm; *p* = 0.01) without supplementary modification of wall thickness.

**Table 6:** **Echocardiographic evaluations of patients with isolated tricuspid repair, subset analysis in Ebstein population (*n* = 14)**.

Characteristic	Preoperative	Follow-up	*p*-Value
Reduced left ventricular ejection fraction (mod-severe)	2 (14.3%)	0	NA
LVIDD (cm)	3.64 ± 0.4	4.0 ± 0.50	0.01
LVISD (cm)	2.41 ± 0.49	2.59 ± 0.43	0.09
IVSD (cm)	0.87 ± 0.18	0.92 ± 0.11	0.28
PWD (cm)	0.88 ± 0.13	0.91 ± 0.11	0.53
RV failure (mod-severe)	7 (50%)	2 (14.3%)	0.47
RV dilatation	14 (100%)	6 (42.8%)	<0.01
Right ventricular internal diastolic diameter (cm)	5.54 ± 1.17	4.28 ± 1.08	*0.03*
LA area (cm^2^)	17.3 ± 1.9	19.6 ± 6.5	0.29
RA area (cm^2^)	48.3 ± 11.2	26.0 ± 10.5	<0.01
Tricuspid regurgitation			
Severe	14 (100%)	0	NA
Haepatic veins backflow	6(42.8%)	2 (14.3%)	0.47
PAP (mmHg)	37.8 ± 5.05	33.3 ± 3.9	0.06
TAPSE (mm)	2.2 ± 1.04	1.35 ± 0.39	<0.01
P.Hypertension (mod-severe)	7(50%)	2 (14.3%)	<0.48

*Data are presented as mean ± SD, median (range), or *n* (%)*.

*LV, left ventricle; RV, right ventricle; LVIDD, left ventricular internal dimension in diastole; LVISD, left ventricular internal end-systolic dimension; IVSD, interventricular septum in diastole; PWD, posterior wall dimensions; RV, right ventricle; LA, left atrium; RA, right atrium; PAP, pulmonary arterial pressure; TAPSE, tricuspid annular plane systolic excursion; P.Hypertension, pulmonary hypertension*.

*Data are expressed as number of events and percentages or otherwise expressed*.

Similar to the main analysis, this sub group showed a substantial reduction in right ventricular diameters (5.54 ± 1.17 vs 4.28 ± 1.08 cm; *p* = 0.03) and RV dilatation (100 vs 42.8%; *p* < 0.01).

An interesting reduction has been observed on right atrial area (48.3 ± 11.2 vs 26 ± 10.5 cm^2^; *p* < 0.01). Furthermore, the Ebstein population also showed a visible reduction of valvular regurgitation between the preoperative and the FU (100 vs 0%; *p*: NA). In addition, TAPSE values were considerably reduced (2.2 ± 1.04 vs 1.35 ± 0.39 mm; *p* < 0.01).

#### Survival

**Figure 2:**
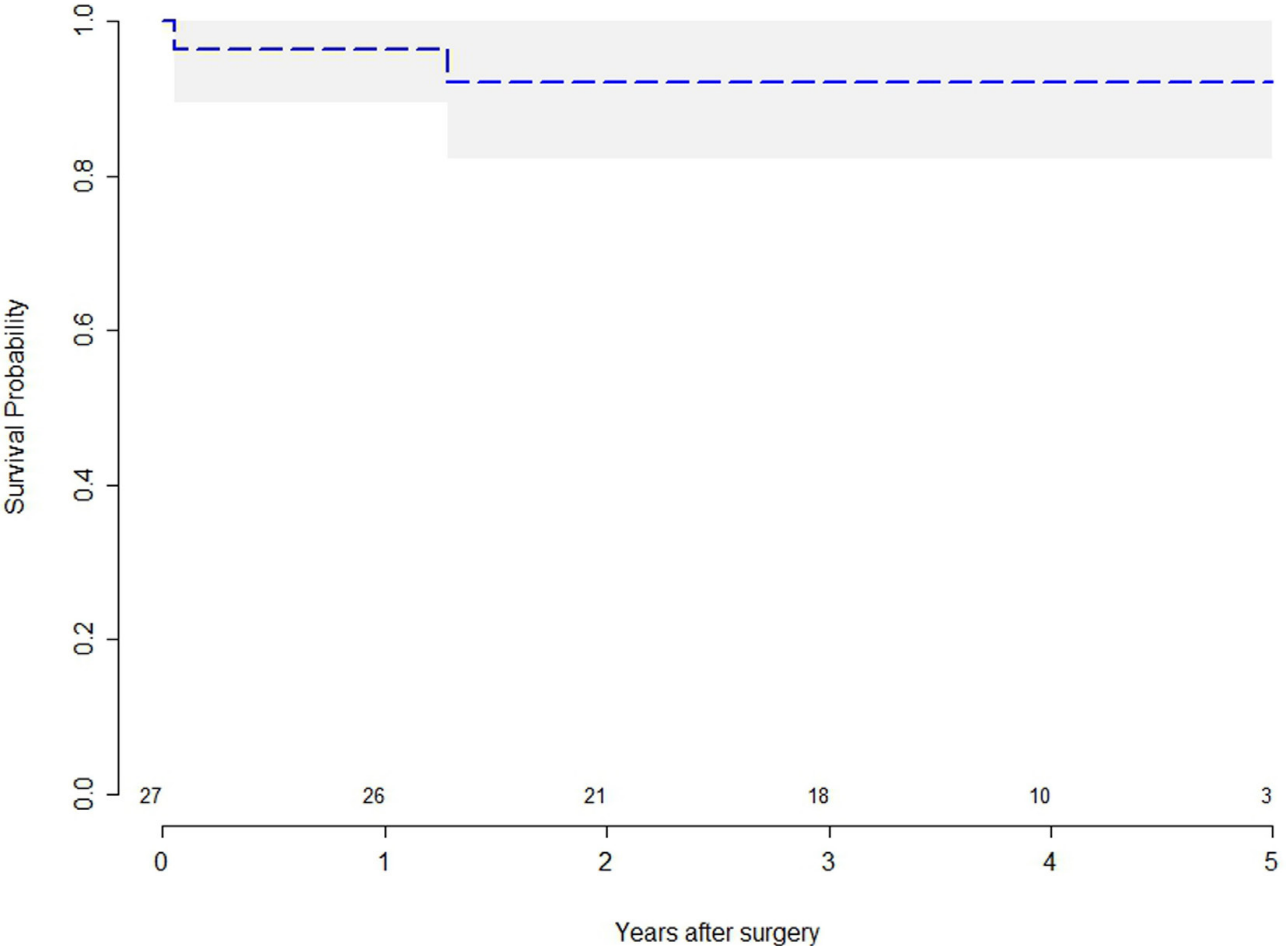
**Kaplan–Meier survival curves for patients with tricuspid regurgitation after tricuspid valve repair**.

Figure 2 reports the survival after surgery. Early mortality shows that one patient (3.7%) died during the admission due to infection of the surgical site (mediastinitis). One patient died, during the FU, from non-cardiac related disease (neoplastic disease). The long-term survival was 96.3% at 1 year and 93.7% at 5 years.

All but one patient remained free from reoperation during the FU.

## Discussion

The incidence of isolated TR among congenital heart disease patients is quite rare, being reported approximately around 1–2% in the Dutch nationwide Congenital Corvitia registry (10). Although previously long underappreciated, TV disease is nowadays receiving increasing attention. However, guidelines for surgical management of tricuspid disease are less aggressive and more subjective than those related to left-sided cardiac valves (3, 11), thus implying that the indications to surgical intervention and methods of approach and repair are not uniform across institutions (7, 12, 13). Interestingly, some studies report no significant differences in outcome among the different surgical strategies (2), whereas other series showed better outcomes of repair vs replacement (1).

Congenital TV disease requiring surgical correction remains a rare entity: we found that, of the TV operations performed over 10 years at our institute, primary isolated tricuspid disease in ACHD patients represented only 8.8% of cases. Previous clinical reports suggest that surgery is beneficial in terms of improvements of symptoms and functional status (6). As reported by Nath et al. (5), improvement in TR severity, regardless of LVEF or PAP, is associated with better survival, while the presence of a severe TR is associated with a poor prognosis (6). Our results showed a significant reduction of TV regurgitation at FU with consequent reduction in haepatic veins flow and mean PAP. Furthermore, patients had a significant improvement in clinical status and reduction of clinical signs of heart failure, thus leading to a significant improvement in quality of life. These findings are in keeping with previous reports showing clinical improvements after TV repair as described by Kim et al. (6).

The 30-day mortality rate also appears to be low in our series (2): one patient died of a surgery-related cause while another patient had a non-cardiac-related death: similar results were demonstrated by previous studies where the operative mortality rate was calculated at 4% (14). The mid-term and long-term survival rates are also reassuring.

The optimal timing for surgical intervention is still debated (10, 15), but in daily practice patients are referred to surgery mainly because of symptoms secondary to RV impairment (1, 10, 15). Our findings were consistent with these data, as patients were referred to surgery at a late stage in the disease course, when highly symptomatic: more than two thirds (67%) were in NYHA class III or IV and clinical signs of right heart failure were reported in 30% of patients. RV failure was reported in 48% of patients and RV dilatation in 81%. Surgical intervention had a significant impact on the remodelling of the RV as shown in our 3-year FU. Echocardiography showed a reduction in RV dimensions (5.15 ± 1.2 vs 4.32 ± 1.16 cm; *p* < 0.01), and RA area (44.7 ± 16.7 vs 26.7 ± 9.2 cm^2^; *p* < 0.01); this is most likely due to the effect of reduced RV pre-load. On the other hand, there was no significant change in RV function (*p* = 0.1); this is probably due to advanced structural modification of the RV which may have occurred before the time of the surgery (16). Therefore, the timing of surgery may be crucial to prevent damage to the RV and to achieve better results in the long term.

Annular repair also leads to increased stability of the cardiac skeleton, which might partially explain the reduction of the TAPSE at FU (1.91 ± 0.85 vs 1.26 ± 0.36 cm; *p* < 0.01).

There was a marked improvement in left ventricular function (14.8 vs 7.4%; *p* < 0.01) despite there being no changes in the left ventricular diameters and wall thickness; possibly due to a reduction of volume of the RV which decreases the compression on the left ventricle.

After performing a sub-analysis of the echocardiographic findings in the Ebstein disease group, we have interestingly noted that the FU findings were in keeping with the results of the whole population analysed.

The incidence of arrhythmia did not change at FU (*p* < 0.1), strengthening the idea that structural damage is a non-reversible phenomenon. As reported in the literature, AF has been recognised as an objective predictor of survival, rising mortality and morbidity due to thromboembolism risk and heart failure (17), making the argument that indication for surgery should be based on RV volumes and function, and leading to surgical intervention before RV failure (6, 12). The right cavities overload reduction leads to a significant clinical benefit, freedom from symptoms and signs of heart failure, and an improvement in quality of life.

For these reasons, we believe that surgical treatment of TR in congenital heart disease should be performed before the onset of heart failure. This concept has already been supported by other studies (1, 2, 6, 12).

Among the cohort of ACHD patients, the RV has also been defined as “forgotten” (12), even though, in the last decade, increasing attention has been focussed on it, in an effort to avoid the adverse outcomes associated with its dysfunction and fibrosis (16). These include not only exercise limitation but also malignant ventricular arrhythmias (17). In various clinical scenarios, TV surgery, as well as pulmonary surgery, is performed earlier in an attempt to “save” the RV, and the surgical mortality in experienced centres is acceptably low (12, 18).

As reported by Warnes (12), the conventional echocardiographic evaluations focussed on the functional assessment of the RV are very challenging because of its complex structure. In contrast, Cardiac Magnetic Resonance and the improved 3D echocardiographic imaging offer better evaluation of the right chambers of the heart (19–21). For these reasons, ACHD should be referred to specific centres in which adequate imaging and better understanding of the pathology can improve assessment and optimise surgical timing when appropriate.

Our study is a retrospective analysis of a relatively small cohort of patients, due to the rarity of isolated TR in ACHD, further multi-centric studies on a larger population should be performed to confirm and expand our findings. The cohort of analysis of this study is also too small to draw a conclusion on long-term re-TV rate.

## Conclusion

Tricuspid valve repair is efficient and durable for the majority of patients with isolated TV regurgitation.

The medium and long-term evaluation show good results on durability and functionality.

When operated at optimal timing, TV repair allows a volume reduction of right cavities associated with both atrial and ventricular reverse remodelling at long-term FU. This condition leads to an improvement in quality of life due to the freedom from symptoms of heart failure.

However, in ACHD populations, these benefits are mostly represented when TV repair intervenes once RV structural damage has not already occurred.

## Informed Consent

The data were derived from previous audits conducted on AHCD patients. The study was conducted in accordance with the principles of the Declaration of Helsinki. The local audit committee approved the study, and the requirement for individual patient consent was waived.

## Author Contributions

Substantial contributions to the conception or design of the work; or the acquisition, analysis, or interpretation of data for the work; drafting the work or revising it critically for important intellectual content; final approval of the version to be published; and agreement to be accountable for all aspects of the work in ensuring that questions related to the accuracy or integrity of any part of the work are appropriately investigated and resolved: RM, VB, PC, GG, FR, AB, UB, and MC.

## Disclaimer

This article/paper/report presents independent research funded by the National Institute for Health Research (NIHR). The views expressed are those of the author(s) and not necessarily those of the NHS, the NIHR or the Department of Health.

## Conflict of Interest Statement

The authors declare that the research was conducted in the absence of any commercial or financial relationships that could be construed as a potential conflict of interest.
